# *MTHFR* Glu429Ala and *ERCC5* His46His Polymorphisms Are Associated with Prognosis in Colorectal Cancer Patients: Analysis of Two Independent Cohorts from Newfoundland

**DOI:** 10.1371/journal.pone.0061469

**Published:** 2013-04-23

**Authors:** Amit A. Negandhi, Angela Hyde, Elizabeth Dicks, William Pollett, Banfield H. Younghusband, Patrick Parfrey, Roger C. Green, Sevtap Savas

**Affiliations:** 1 Discipline of Genetics, Faculty of Medicine, Memorial University of Newfoundland, St. John’s, Newfoundland, Canada; 2 Clinical Epidemiology Unit, Faculty of Medicine, Memorial University of Newfoundland, St. John’s, Newfoundland, Canada; 3 Discipline of Surgery, Faculty of Medicine, Memorial University of Newfoundland, St. John’s, Newfoundland, Canada; 4 Discipline of Oncology, Faculty of Medicine, Memorial University of Newfoundland, St. John’s, Newfoundland, Canada; MOE Key Laboratory of Environment and Health, School of Public Health, Tongji Medical College, Huazhong University of Science and Technology, China

## Abstract

**Introduction:**

In this study, 27 genetic polymorphisms that were previously reported to be associated with clinical outcomes in colorectal cancer patients were investigated in relation to overall survival (OS) and disease free survival (DFS) in colorectal cancer patients from Newfoundland.

**Methods:**

The discovery and validation cohorts comprised of 532 and 252 patients, respectively. Genotypes of 27 polymorphisms were first obtained in the discovery cohort and survival analyses were performed assuming the co-dominant genetic model. Polymorphisms associated with disease outcomes in the discovery cohort were then investigated in the validation cohort.

**Results:**

When adjusted for sex, age, tumor stage and microsatellite instability (MSI) status, four polymorphisms were independent predictors of OS in the discovery cohort *MTHFR* Glu429Ala (HR: 1.72, 95%CI: 1.04–2.84, p = 0.036), *ERCC5* His46His (HR: 1.78, 95%CI: 1.15–2.76, p = 0.01), *SERPINE1 −*675indelG (HR: 0.52, 95%CI: 0.32–0.84, p = 0.008), and the homozygous deletion of *GSTM1* gene (HR: 1.4, 95%CI: 1.03–1.92, p = 0.033). In the validation cohort, the *MTHFR* Glu429Ala polymorphism was associated with shorter OS (HR: 1.71, 95%CI: 1.18–2.49, p = 0.005), although with a different genotype than the discovery cohort (CC genotype in the discovery cohort and AC genotype in the validation cohort). When stratified based on treatment with 5-Fluorouracil (5-FU)-based regimens, this polymorphism was associated with reduced OS only in patients not treated with 5-FU. In the DFS analysis, when adjusted for other variables, the TT genotype of the *ERCC5* His46His polymorphism was associated with shorter DFS in both cohorts (discovery cohort: HR: 1.54, 95%CI: 1.04–2.29, p = 0.032 and replication cohort: HR: 1.81, 95%CI: 1.11–2.94, p = 0.018).

**Conclusions:**

In this study, associations of the *MTHFR* Glu429Ala polymorphism with OS and the *ERCC5* His46His polymorphism with DFS were identified in two colorectal cancer patient cohorts. Our results also suggest that the *MTHFR* Glu429Ala polymorphism may be an adverse prognostic marker in patients not treated with 5-FU.

## Introduction

Colorectal cancer has a high incidence in the developed countries [1]. In 2004 this disease was the 4^th^ leading cause of death due to cancer with over 600,000 deaths worldwide [2]. In Canada, it is a major health concern with an estimated 22,200 new cases and 8,900 deaths expected in 2011 [3]. There are significant inter-provincial variations in incidence and mortality rates, and the province of Newfoundland and Labrador (NL) has the highest age-standardized incidence and mortality rates for colorectal cancer among the Canadian provinces [3]. Both genetic and environmental factors play a role in susceptibility to colorectal cancer. While the majority of the colorectal cancer patients are sporadic cases, nearly 5% of the colorectal cancers are caused by inherited high-penetrant mutations [4]. Thirty-five per cent of the risk for developing sporadic colorectal cancer is also attributed to the inherited factors [5].

Important colorectal cancer outcomes include recurrence, metastasis and death. Currently, the most valuable prognostic criterion in colorectal cancer patients is the TNM (tumor-node-metastasis) staging defined by the American Joint Committee on Cancer [6]. Generally, patient prognosis worsens with increasing stage.

A number of clinical and molecular parameters have also been investigated for their prognostic utility in colorectal cancer. For instance, Popat et al [7] reported in their meta-analysis that patients with microsatellite instability-high (MSI-H) tumors have a more favorable prognosis when compared to patients with microsatellite instability-low (MSI-L) or microsatellite stable (MSS) tumors. Several other clinicopathological and molecular features have also been reported to be associated with prognosis, such as high tumor grade [8], mucinous histology [9], lymphovascular invasion [10], chromosomal instability [11], and the presence of the *BRAF1* Val600Glu somatic mutation in tumors [12], though contradictory reports have also been published [13]–[15]. Inconsistent results on the association between familial risk status and survival of colorectal cancer patients were also reported [16], [17]. Additionally, demographic factors such as gender and ethnicity may be modifiers of prognosis [6]. These factors only partly account for the variations in cancer patient outcomes and it is possible that genetic factors (such as single nucleotide polymorphisms (SNPs), insertion/deletion (indel) polymorphisms, and somatic mutations) may influence prognosis. Their investigation thus may help understanding the reasons for the inter-patient outcome variability and the underlying biological mechanisms.

Several studies have previously reported significant associations between genetic variations and outcomes in colorectal cancer patients. In the present study, we investigated 27 such polymorphisms (**Table 1**) as potential prognostic factors in a colorectal cancer patient cohort (discovery cohort, n = 532) and subsequently tested the validity of the positive associations in an additional colorectal cancer patient cohort (validation cohort, n = 252).

**Table 1:** Polymorphisms investigated in the discovery cohort.

Pathway	Gene	Polymorphism	SNP ID	Minor alleleand MAF inthe discoverycohort(replicationcohort)	****MAF(Caucasian)	Chromosome,location	Functional impact of the polymorphism
Cell cycle	*CCND1*	Pro241Pro, A/G	*rs9344	A, 45.28%	48–63%	Chr 11, 69462910	G allele causes an alternative transcript of *CCND1* mRNA [55]
Cell adhesion & signaling	*DCC*	Arg201Gly, C/G	*rs2229080	G, 36.98%	33–42%	Chr 18, 50432602	G allele associated with reduced gene expression [56]
Cell signaling	*EGFR*	Arg521Lys, G/A	*rs2227983	A, 26.89%	22–30%	Chr 7, 55229255	A allele associated with reduced ability of EGFR to induce cell growth [57]
	*FGFR4*	Gly388Arg, A/G	*rs351855	T, 31.26%	26–31%	Chr 5, 176520243	G allele associated with greater motility and progression of cancer cells [58]
DNA repair	*ERCC1*	Asn118Asn, C/T	*rs11615	C, 37.57%	33–45%	Chr 19, 45923653	T allele associated with reduced gene expression [59]
	*ERCC2*	Lys751Gln, G/T	*rs13181	G, 35.69%	27–42%	Chr 19, 45854919	G allele linked with inefficient DNA repair [60]
	*ERCC5*	His46His, C/T	*rs1047768	T, 41.13% (42.15%)	32–51%	Chr 13, 103504517	unknown
	*EXO1*	Pro757Leu, C/T	*rs9350	T, 14.6%	15–27%	Chr 1, 242048674	unknown
	*OGG1*	Ser326Cys, C/G	*rs1052133	G, 23.54%	15–22%	Chr 3, 9798773	G allele reduces DNA binding and repair activity [61]
	*MLH1*	Ile219Val, A/G	*rs1799977	G, 28.63%	0–35%	Chr 3, 3705356	unknown
	*XRCC1*	Arg399Gln, G/A	*rs25487	A, 34.36%	37–58%	Chr 19, 44055726	A allele associated with impaired DNA repair function [62]
	*XRCC3*	Thr241Met, C/T	*rs861539	T, 39.74%	37–65%	Chr 14, 104165753	T allele associated with defective DNA repair mechanism [63]
Apoptosis	*FAS*	c.-24+733T>C	*rs1800682	C, 44.91%	39–50%	Chr 10, 90749963	unknown
Drug metabolism	*GSTM1*	gene deletion	**na	non-deletion allele, 45.10% (44.54%)	*****38–62%		loss of gene function
	*GSTP1*	Ile105Val, A/G	*rs1695	G, 36.67%	29–42%	Chr 11, 67352689	G allele associated with reduced enzymatic activity [64]
	*GSTT1*	gene deletion	**na	deletion allele, 17%	*****15–20%		loss of gene function
Inflammation	*IL6*	−174G/C in promoter	*rs1800795	C, 44.25%	50–57%	Chr 7, 22766645	C allele linked with reduced gene expression [65]
	*PTGS2*	c.3618A/G in 3′-UTR	*rs4648298	G, 1.63%	1.7–1.8%	Chr 1, 186641682	unknown
Tissue remodeling	*MMP1*	−1607 indel G inpromoter	***rs1799750	G, 46.9%	43.30%	Chr 11, 102670496	insG allele associated with enhanced gene transcription [66]
	*MMP2*	−1306C/T in promoter	*rs243865	T, 22.92%	18–25%	Chr 16, 55511806	T allele associated with reduced gene expression [67]
DNA synthesis	*MTHFR*	Ala222Val, C/T	*rs1801133	T, 31.77%	21–37%	Chr 1, 11856378	T allele associated with reduced protein activity [68] and amount [69]
	*MTHFR*	Glu429Ala, A/C	*rs1801131	C, 30.61% (30%)	33–38%	Chr 1, 11854476	C allele associated with reduced enzymatic activity [31]
	*TYMS*	2R/3R in 5′-UTR	**rs34743033	2R, 46.6%	44.60%	Chr 18, 657646∶657730	3R allele confers increased translational efficiency [70]
	*TYMS*	indel 6 bp in 3′-UTR	*rs16430	del, 34.13%	37.00%	Chr 18, 673444	deletion of 6 bp associated with lower mRNA stability [71]
Angiogenesis	*SERPINE1*	−675 indelG inpromoter	***rs1799889	G, 46.71% (46.53%)	54.30%	Chr 7, 100769710∶100769711	insG allele linked with lower transcriptional activity [72]
	*VEGFA*	−634G/C in 5′-UTR	*rs2010963	C, 29.1%	20–43%	Chr 6, 43738350	G allele associated with low gene expression [73]
	*VEGFA*	+936C/T in 3′-UTR	*rs3025039	T, 10.73%	10–22%	Chr 6, 43752536	T allele associated with lower plasma VEGF levels [74]

na: not available, MAF: minor allele frequency, VNTR: variable number of tandem repeats, 2R: 2 VNTR repeats, 3R: 3 VNTR repeats. The *EGFR* rs2227983 polymorphism is also known as rs11543848. The *PTGS2* c.3618A/G excluded from analysis due to its low minor allele frequency.

* genotyped by MassArray® technology,

** genotyped by gel electrophoresis of PCR-amplified fragments,

*** genotyped by TaqMan® SNP genotyping assays.

**** MAF information was retrieved from the dbSNP database [75].

***** MAFs are as reported in a published report [76]. The chromosomal locations of polymorphisms are extracted from the dbSNP database [75] (Genome Reference Consortium Human Build 37 patch release 5).

## Materials and Methods

### Ethics Statement

This study includes two patient cohorts. For both cohorts, collection of the patient clinical data and biospecimens was approved for research purposes by the Regional Health Boards and the Human Investigation Committee (HIC) of Memorial University of Newfoundland. In the discovery cohort, written informed consent was obtained from the patients recruited or their proxies. The Human Investigation Committee of Memorial University of Newfoundland waived the need for written informed consent from the participants in the replication cohort. Ethics approval for this particular project was also obtained from the Human Investigation Committee of Memorial University of Newfoundland.

### Patient Cohorts

#### a) The discovery cohort

This cohort consisted of 532 colorectal cancer patients from the Newfoundland Colorectal Cancer Registry (NFCCR). NFCCR was established in 1999 and recruited 736 stage I–IV colorectal cancer patients between 1999 and 2003 [18]. All patients were ≤75 years old and their diagnosis was confirmed by pathological examination. The molecular and genetic characteristics of this cohort and other details have been previously reported by others [19], [20]. For all patients, the clinical data was compiled (although there were also missing values for some variables; **Table 2**). In this study, 532 of the 736 colorectal cancer patients from the NFCCR were investigated for whom the genomic DNA (extracted from blood) was also available. Patient data on clinicopathological features, recurrence and metastasis, and the date of death were retrieved from clinical reports (medical, pathology, radiology, autopsy, and surgical reports, lab investigations, physicians’ assessment and progress notes, inpatient discharge summaries), the Newfoundland Cancer Treatment and Research Foundation database, or patient follow-up questionnaires. In this cohort, 62% of the patients were treated with 5-FU based chemotherapy in either neoadjuvant or adjuvant settings or upon diagnosis of local and distant recurrences, whereas the remaining patients were either not treated with chemotherapy, or were treated with cisplatin/etoposide (n = 1). Patients in this cohort were followed until April 2010. The median follow-up time in this cohort for overall survival and disease free survival was 6.4 and 6 years, respectively (**Table 2**).

**Table 2:** Baseline characteristics of the discovery and the validation cohorts.

Variable	Discovery cohort n (%)	Validation cohort n (%)	p-value
**Sex**			
Male	327 (61.50%)	133 (52.78%)	
Female	205 (38.50%)	119 (47.22%)	**p = 0.021**
**Median age in years (range)**	61.4 (20.7–75)	68.7 (25.3–91.6)	**p<0.001**
**Histology**			
non-mucinous	471 (88.50%)	211 (83.73%)	
Mucinous	61 (11.50%)	41 (16.27%)	p = 0.062
**Location**			
Colon	353 (66.40%)	202 (80.16%)	
Rectum	179 (33.60%)	50 (19.84%)	**p<0.001**
**Stage**			
I	99 (18.60%)	48 (19.05%)	
II	206 (38.70%)	88 (34.92%)	
III	175 (32.90%)	68 (26.98%)	
IV	52 (9.80%)	41 (16.27%)	
Unknown	–	7 (2.78%)	**p = 0.034**
**Grade**			
well diff./moderately diff.	489 (91.90%)	211 (83.73%)	
poorly diff./undiff.	39 (7.30%)	37 (14.68%)	
Unknown	4 (0.80%)	4 (1.59%)	**p = 0.001**
* **Invasion**			
Absence	326 (61.30%)	64 (25.40%)	
Presence	166 (31.20%)	101 (40.08%)	
Unknown	40 (7.50%)	87 (34.52%)	**p<0.001**
**OS status**			
Dead	177 (33.30%)	155 (61.51%)	
Alive	354 (66.60%)	97 (38.49%)	
Unknown	1 (0.10%)	–	**p<0.001**
**Median OS follow-up time in years (range)**	6.4 (0.4–10.9)	5.4 (0–12.48)	
**DFS status**			
Event	208 (39.10%)	167 (66.27%)	
no event	323 (60.71%)	85 (33.73%)	
Unknown	1 (0.19%)	–	**p<0.001**
**Median DFS follow-up time in years (range)**	6 (0.2–10.9)	3.3 (0–12.5)	
**MSI Status**			
MSI-H	56 (10.50%)	24 (9.52%)	
MSI-L/MSS	455 (85.50%)	228 (90.48%)	
Unknown	21 (4%)		p = 0.543
**Familial risk**			
Low	256 (48.10%)	Na	nd
Intermediate/high	276 (59.10%)		
***BRAF1*** ** Val600Glu mutation**			
Presence	49 (9.20%)	Na	nd
Absence	435 (81.80%)		
Unknown	48 (9%)		
**5-FU based treatment**			
5-FU treated	330 (62.03%)	88 (34.92%)	
other/no chemotherapy	199 (37.41%)	148 (58.73%)	
Unknown	3 (0.56%)	16 (6.35%)	**p<0.001**

* Vascular invasion and lymphatic invasion were highly correlated in the discovery cohort (p<0.001). Since data for vascular invasion in the validation cohort was not available, vascular invasion in the discovery cohort and lymphatic invasion in the validation cohort were compared with each other. diff: differentiated, n: number of patients, na: not available, nd: not done.

#### b) The validation cohort

This is a retrospective cohort comprised of 280 previously collected colorectal cancer patients from the Avalon Peninsula of Newfoundland. For all 280 patients the clinical data was collected, however genomic DNA (extracted from non-tumor tissues) was available for only 252 patients. Hence, 252 of 280 patients were included into the present study. Patients in this cohort were diagnosed with primary colorectal cancer in a two-year period (between 1997–1998). The patient selection criteria are as follows: a) patients with carcinoma in polyp were included only if the tumor invaded into the stalk, b) patients whose colorectal cancer was a recurrence of an earlier colorectal cancer or a metastasis from a distant organ, and those with carcinoid tumors, familial adenomatous polyposis, carcinoma in situ and mucosal carcinoma were excluded, and c) patients were selected regardless of their age of diagnosis. Prognostic data of these patients was collected from the medical and hospital records and the Newfoundland and Labrador Centre for Health Information. In the validation cohort, 34.9% of the patients were treated with 5-FU-based chemotherapy in either neoadjuvant or adjuvant settings or upon diagnosis of local or distant recurrences. The remaining patients were either not treated with chemotherapy or were treated with other agents such as irinotecan, tomudex or oxaliplatin. Patients in this cohort were followed until July 2009. The median follow-up time for this cohort was 5.4 and 3.3 years for overall survival and disease free survival, respectively (**Table 2**).

### Selection of Polymorphisms

The dbCPCO database [21] (http://www.med.mun.ca/cpco/) summarizes literature on genetic markers studied for their prognostic associations in colorectal cancer patients. In August 2010, a search of the entries in this database for survival measures (e.g. overall survival) was performed. As a result of this search, 31 polymorphisms were identified. Out of 31, one polymorphism (*EGFR* (CA)_n_ repeat) was not included in this study because of the lack of a suitable equipment in our lab required to obtain its genotypes. In addition, three polymorphisms (*EGF* A61G, *TP53* Arg72Pro and *PTGS2 −*765 G/C) could not be genotyped using the MassArray® technology. As a result, 27 polymorphisms from 24 different genes that were a) reported to be associated with overall survival in at least one study (either univariate or multivariate analyses) (**Table S1** in **File S1**), b) suitable to be genotyped using the genotyping techniques used in this project (e.g. single nucleotide polymorphisms, indels, microsatellite repeats, gene deletions), and c) successfully genotyped (i.e. did not fail to be genotyped using the MassArray® method) were investigated in the current study (**Table 1**).

### Genotyping Methods

The genotypes for 27 polymorphisms were obtained in the discovery cohort and the genotypes for four polymorphisms that were associated with OS in the discovery cohort (*MTHFR* Glu429Ala, *ERCC5* His46His, *SERPINE1 −*675indelG, and *GSTM1* gene deletion) were obtained in the validation cohort. Genotypes were obtained using the Sequenom MassArray® platform, TaqMan® SNP genotyping assays and gel electrophoresis of PCR-amplified fragments. Further details related to genotyping experiments can be found in the **Methods S1** and the **Table S2** in **File S1**. Each genotyping reaction included non-template amplifications as negative controls. At least 5.9% of the genotypes were successfully duplicated with a minimum 99.7% concordance rate. Samples with discordant genotypes were either re-genotyped (genotypes obtained by using the TaqMan® SNP genotyping assays and gel electrophoresis of PCR-amplified fragments) or excluded from analysis (genotypes obtained by using the Sequenom MassArray® technique). The minimum successful genotyping rates were 97.4% for the discovery cohort and 94.44% for the validation cohort. In the case of in-house genotyping experiments (i.e. TaqMan® SNP genotyping assays and the gel-electrophoresis of PCR-amplified fragments), genotyping reactions for failed DNA samples were attempted at least two additional times, depending on the availability of DNA.

### Statistical Analyses

#### a) Hardy Weinberg Equilibrium (HWE) test

HWE test was manually performed for polymorphisms using the Pearson’s Chi-square test (**Table S3** in **File S1**). For the *GSTM1* and *GSTT1* gene deletions HWE was not tested as heterozygote genotypes cannot be detected using the genotyping methodology applied in this study.

#### b) Survival endpoints

OS time was the time from diagnosis of colorectal cancer until death from any cause. DFS time was the time from diagnosis of colorectal cancer until the occurrence of metastasis, recurrence or death from any cause, whichever was earlier. Patients who did not experience the outcome of interest were censored at the time of last follow up.

#### c) Variables

Categorical variables analyzed were sex (males vs females), tumor histology (mucinous vs non-mucinous), tumor location (rectal vs colon), stage (stages II, III and IV vs stage I), tumor grade (poorly differentiated/undifferentiated vs well/moderately differentiated), vascular and lymphatic invasions (present vs absent), familial risk (high/intermediate risk vs low risk), microsatellite instability status (MSI-H vs MSI-L/MSS) and *BRAF1* Val600Glu mutation status (present vs wildtype). For the discovery set, the familial risk status was determined previously by the NFCCR investigators using the Amsterdam II and revised Bethesda criteria [18]. Tumor MSI status and *BRAF1* Val600Glu status analyses were also previously performed by NFCCR [19], [20], [22]. Vascular and lymphatic invasions were highly correlated in the discovery cohort (>95% of tumors with vascular invasion also had lymphatic invasion). Please note that in some models, confidence intervals for the stage IV patients were wide, reflecting the small sample size for this group of patients. These results therefore should be interpreted cautiously.

We categorized the genotypes for each polymorphism assuming the co-dominant genetic model (i.e. minor allele homozygotes and heterozygotes were individually compared to the major allele homozygotes). In the case of the *MTHFR* Glu429Ala polymorphism, we also performed multivariable analyses under the recessive (CC vs CA+AA genotypes) and the dominant (CC+CA vs AA genotypes) genetic models. The *PTGS2* c.3618A/G polymorphism was excluded from the statistical analysis due to its very low minor allele frequency (1.63%). For the VNTR polymorphism in the *TYMS* gene (rs34743033), 0.93% of the samples in the discovery cohort had a rare 4R allele. These patients were combined with 3R/3R genotypes for analyses. Age was the only continuous variable in our analysis.

#### d) Univariate analyses

Time-to-event survival plots were constructed using the Kaplan-Meier method and were compared by the log-rank test.

#### e) Multivariable analysis

The variables used in the construction of the final multivariable models were selected by backward elimination method for OS and DFS separately using the Cox regression method. Selected variables were then re-entered in the final models. The proportionality assumption was verified by examining the log-minus-log (log(-log(S(t)))) plots. We also tested the interaction between the *MTHFR* Glu429Ala genotypes (co-dominant genetic model) and the 5-FU treatment using the Cox regression method.

#### f) Stratified analyses

Since the MTHFR enzyme (thus the Glu429Ala polymorphism by modifying the MTHFR enzymatic activity) plays a biological role in the 5-FU metabolism/efficacy (See Discussion), 5-FU stratification was done only for this polymorphism. Since this polymorphism was not associated with DFS, 5-FU stratification analysis for DFS was not performed.

#### g) Comparisons of cohorts

To test if the differences between the baseline characteristics of the discovery and the validation cohorts were significant, we performed the Chi-square test for the categorical variables. Since age was not normally distributed in both cohorts, the non-parametric Mann Whitney-U test was used to compare differences in distribution of age between these two cohorts. Similar analyses were also performed to compare the entire NFCCR cohort (n = 736) and the discovery cohort (n = 532) and also the entire second cohort (n = 280) and validation cohort included in our analysis (n = 252).

PASW Statistics 18 software release 18.0.2 (IBM, NY, USA) was used to perform the statistical analyses. All tests were double sided and the significance threshold was set at p = 0.05. To avoid false-negative results, correction for multiple testing was not performed in the discovery cohort analysis. While this also increases the potential number of false-positive associations, analysis of the associations detected in the discovery cohort in an additional patient cohort (i.e. the replication cohort) helped eliminate the false-positive findings.

## Results

### The Discovery Cohort Characteristics

Baseline characteristics of the discovery cohort are listed in **Table 2**. The median age at diagnosis was 61.4 years. One-third (33.3%) of the patients had died and 39% of patients had experienced recurrence, metastasis or death by the time of last follow-up. The discovery cohort has a significantly lower proportion of stage IV patients (9.8%) when compared to the entire NFCCR cohort (20.9%) (p<0.001). The discovery cohort also significantly differed from the NFCCR cohort in terms of proportions of tumors with vascular (p = 0.007) and lymphatic invasions (p = 0.021), and deceased patients (p<0.001).

### Overall Survival Analysis in the Discovery Cohort

Out of 26 polymorphisms investigated, four polymorphisms were significantly associated with OS when adjusted for sex, age, stage and MSI status (**Table 3**). Briefly, for the *MTHFR* Glu429Ala polymorphism, patients homozygous for the C allele had shorter survival (HR: 1.72, 95% CI: [1.04–2.84], p = 0.036) compared to patients homozygous for the A allele. For the *ERCC5* His46His polymorphism, patients with the TT genotype had a greater risk of death (HR: 1.78, 95% CI: [1.15–2.76], p = 0.01) compared to those patients with the CC genotype. In the case of the *SERPINE1 −*675indelG polymorphism, the minor allele homozygotes (insG/insG) had increased survival (HR: 0.52, 95% CI: [0.32–0.84], p = 0.008) compared to the patients with delG/delG genotype. Genotype distributions of these three polymorphisms were in HWE (p>0.05; **Table S3** in **File S1**). In addition, patients with at least one copy of *GSTM1* gene had a greater risk of death compared to patients with no copy of the gene (HR: 1.40, 95% CI: [1.03–1.92], p = 0.033) (**Table 3**).

**Table 3:** Multivariable Cox regression analysis results for overall survival in the discovery and validation cohorts.

	Discovery cohort (n = 504, deaths = 168)			Validation cohort (n = 224, deaths = 134)		
*Co-dominant genetic model						
*#*Variable	HR (95% CI)	p-value	n	HR (95% CI)	p-value	n
***MTHFR*** ** Glu429Ala**		0.105			**0.010**	
CA vs AA	1.18 (0.84–1.64)	0.342	230 vs 232	**1.71 (1.18–2.49)**	**0.005**	92 vs 112
CC vs AA	**1.72 (1.04–2.84)**	**0.036**	42 vs 232	0.89 (0.45–1.74)	0.730	20 vs 112
***ERCC5*** ** His46His**		**0.034**			0.609	
TC vs CC	1.37 (0.94–1.97)	0.098	240 vs 173	1.20 (0.80–1.80)	0.387	112 vs 76
TT vs CC	**1.78 (1.15–2.76)**	**0.010**	91 vs 173	1.26 (0.74–2.16)	0.398	36 vs 76
***SERPINE1*** * −* **675indelG**		**0.029**			0.716	
insG/delG vs delG/delG	0.81 (0.57–1.15)	0.238	258 vs 141	1.19 (0.78–1.80)	0.420	103 vs 69
insG/insG vs delG/delG	**0.52 (0.32–0.84)**	**0.008**	105 vs 141	1.08 (0.67–1.73)	0.766	52 vs 69
***GSTM1*** ** gene deletion**						
present vs absent	**1.40 (1.03–1.92)**	**0.033**	228 vs 276	1.23 (0.86–1.78)	0.261	99 vs 125
**Sex**						
male vs female	**1.46 (1.04–2.05)**	**0.031**	313 vs 191	1.28 (0.90–1.84)	0.175	118 vs 106
**Age at diagnosis (per year)**	**1.02 (1–1.04)**	**0.046**		**1.05 (1.03–1.07)**	**<0.001**	
**Stage**		**<0.001**			**<0.001**	
II vs I	1.47 (0.84–2.59)	0.180	194 vs 95	1.14 (0.63–2.09)	0.662	80 vs 44
III vs I	**2.08 (1.19–3.64)**	**0.010**	165 vs 95	**2.61 (1.45–4.71)**	**0.001**	64 vs 44
IV vs I	**11.69 (6.45–21.16)**	**<0.001**	50 vs 95	**11.32 (5.92–21.67)**	**<0.001**	36 vs 44
**MSI status**						
MSI-H vs MSI-L/MSS	**0.23 (0.09–0.64)**	**0.004**	56 vs 448	**0.26 (0.11–0.61)**	**0.002**	21 vs 203
** **Dominant genetic model**						
*MTHFR* Glu429Ala	1.19 (0.87–1.61)	0.277	272 vs 232	**1.56 (1.12–2.17)**	**0.009**	122 vs 121
(CA+CC vs AA)						
*** **Recessive genetic model**						
*MTHFR* Glu429Ala	**1.80 (1.13–2.86)**	**0.014**	42 vs 462	0.69 (0.38–1.25)	0.219	21 vs 222
(CC vs CA+AA)						

* The multivariable Cox regression model assuming the co-dominant genetic model contained the *MTHFR* Glu429Ala, *ERCC5* His46His, *SERPINE1 −*675indelG, *GSTM1* gene deletion genotypes as well as sex, age, stage and MSI status as covariates.

** Only the multivariable Cox regression analysis result for the *MTHFR* Glu429Ala polymorphism when adjusted for sex, age, stage and MSI status is shown (assuming the dominant genetic model). The complete multivariable models for the dominant genetic model can be found in **Tables S4** and **S6** in **File S1** for the discovery and validation cohorts.

*** The multivariable Cox regression analysis result for the *MTHFR* Glu429Ala polymorphism when adjusted for sex, age, stage and MSI status is shown (assuming the recessive genetic model). The complete multivariable models can be found in **Tables S5** and **S7** in **File S1** for the discovery and validation cohorts. CI: confidence interval, HR: hazard ratio, n: number of patients, vs: versus.

#The major homozygote genotypes and other referent categories are underlined.

### Disease-free Survival Analysis in the Discovery Cohort

Out of 26 polymorphisms, the *ERCC5* His46His and *OGG1* Ser326Cys polymorphisms were associated with shorter DFS in the discovery cohort when adjusted for other variables (**Table 4**). Specifically, patients homozygous for the T allele of the *ERCC5* His46His (HR: 1.54, 95% CI: [1.04–2.29], p = 0.032) and G allele of the *OGG1* Ser326Cys (HR: 1.81, 95% CI: [1.08–3.04], p = 0.025) had worse survival compared to the major allele homozygotes.

**Table 4:** Multivariable model for disease-free survival in the discovery and validation cohorts.

	Discovery cohort (n = 504, events = 198)			Validation cohort (n = 227, events = 148)		
#Variable	HR (95% CI)	p-value	n	HR (95% CI)	p-value	n
***ERCC5*** ** His46His**		0.098			**0.036**	
TC vs CC	1.24 (0.89–1.72)	0.211	240 vs 172	**1.48 (1.02–2.17)**	**0.041**	114 vs 77
TT vs CC	**1.54 (1.04–2.29)**	**0.032**	92 vs 172	**1.81 (1.11–2.94)**	**0.018**	36 vs 77
***OGG1*** ** Ser326Cys**		0.082		Nd	Nd	
GC vs CC	1.09 (0.80–1.48)	0.590	167 vs 304			
GG vs CC	**1.81 (1.08–3.04)**	**0.025**	33 vs 304			
***ERCC1*** ** Asn118Asn**		0.152		Nd	Nd	
TC vs TT	1.19 (0.87–1.64)	0.281	215 vs 206			
CC vs TT	1.48 (0.99–2.20)	0.054	83 vs 206			
***TYMS*** ** indel 6 bp**		0.171		Nd	nd	
ins 6 bp/del 6 bp vs ins 6 bp/ins 6 bp	0.83 (0.61–1.13)	0.235	226 vs 221			
del 6 bp/del 6 bp vs ins 6 bp/ins 6 bp	1.25 (0.80–1.96)	0.325	57 vs 221			
***GSTM1*** ** gene deletion**						
present vs absent	1.28 (0.96–1.70)	0.090	229 vs 275	1.17 (0.84–1.63)	0.366	101 vs 126
**Location**						
rectum vs colon	1.33 (0.99–1.79)	0.055	166 vs 338	1.07 (0.71–1.60)	0.743	44 vs 183
**Stage**		**<0.001**			**<0.001**	
II vs I	1.51 (0.93–2.47)	0.099	194 vs 95	**1.82 (1.04–3.19)**	**0.036**	81 vs 45
III vs I	**2.09 (1.28–3.41)**	**0.003**	164 vs 95	**3.14 (1.79–5.51)**	**<0.001**	65 vs 45
IV vs I	**6.24 (3.69–10.53)**	**<0.001**	51 vs 95	**130.16 (52.48–322.83)**	**<0.001**	36 vs 45
**MSI status**						
MSI-H vs MSI-L/MSS	**0.35 (0.17–0.71)**	**0.004**	55 vs 449	**0.37 (0.18–0.76)**	**0.007**	22 vs 205

The multivariable model contained location, stage and MSI status in addition to the *ERCC5* His46His, *OGG1* Ser326Cys, *ERCC5* Asn118Asn, *TYMS* indel 6 bp, and *GSTM1* gene deletion genotypes in the discovery cohort and the *ERCC5* His46His and *GSTM1* gene deletion genotypes in the validation cohort as covariates. The genotypes for the *OGG1* Ser326Cys, *ERCC5* Asn118Asn, and *TYMS* indel 6 bp polymorphisms were not available in the validation cohort. CI: confidence interval, HR: hazard ratio, n: number of patients, nd: not done, vs: versus. Event refers to recurrence, metastasis or death in the patient, whichever had occurred earlier.

#The referent categories are underlined. Please note that reflecting the small numbers of patients in the validation cohort, the CIs for the effect estimate in stage IV patients are quite wide and should not be interpreted as an accurate estimation.

### The Validation Cohort Characteristics

Baseline characteristics of the validation cohort are listed in **Table 2**. The median age of diagnosis was 68.7 years. By the time of last follow-up 61.5% of patients had died and 66.3% of the patients had experienced recurrence, metastasis or death. There were no statistically significant differences between the initial 280 patients in this cohort and the 252 patients included in this study in terms of clinical and molecular features (*data not shown*).

However, there were significant differences between the discovery and validation cohorts in terms of clinicopathological characteristics. First, there was a higher proportion of stage IV patients in the validation cohort (16.3%) compared to the discovery cohort (9.8%) (p = 0.034). Second, the median age at diagnosis in the validation cohort (68.7 years) was significantly higher (p<0.001) compared to that of the discovery cohort (61.4 years). The proportions of patients in terms of sex, tumor location, grade, OS and DFS status, vascular and lymphatic invasions, and treatment with 5-FU-based regimens were also different between the two cohorts (**Table 2**).

### Overall Survival Analysis in the Validation Cohort

**Figure 1:**
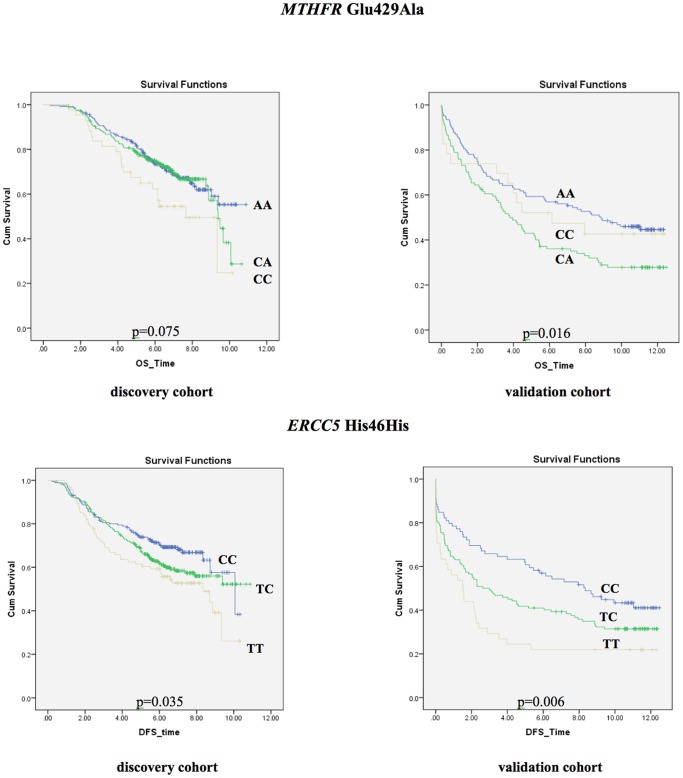
Kaplan-Meier survival curves for the *MTHFR* Glu429Ala (overall survival) and the *ERCC5* His46His polymorphisms (disease-free survival) assuming the co-dominant genetic model. P-values are based on log-rank test.

The genotype distribution of four polymorphisms tested in the validation cohort did not deviate from HWE. Out of these four polymorphisms, only the *MTHFR* Glu429Ala polymorphism was associated with shorter survival times when adjusted for other variables **(** **Table 3** **)**. However, in contrast to the discovery set, in the validation cohort, the heterozygotes (Glu/Ala) had shorter survival (HR: 1.71, 95% CI: [1.18–2.49], p = 0.005) when compared to homozygotes for glutamate (Glu/Glu) (**Figure 1**). Thus the genotype associated with worse survival in the validation cohort (AC) was different than the genotype associated in the discovery cohort (CC). Therefore, we also performed multivariable analyses assuming the recessive and dominant genetic models in these two cohorts (**Table 3**, **Tables S4–S7** in **File S1**). As a result, in the discovery set, the *MTHFR* Glu429Ala polymorphism was associated with OS in the recessive genetic model (CC vs AC+AA; HR: 1.80, 95% CI: [1.13–2.86], p = 0.014), but not in the dominant genetic model. In contrast, in the validation set, this polymorphism was associated with OS in the dominant genetic model (CC+AC vs AA; HR: 1.56, 95% CI: [1.12–2.17], p = 0.009), but not in the recessive genetic model. Analysis of this polymorphism assuming the additive genetic model did not yield significant association with OS in either cohort (*data not shown*). Thus, the association of the *MTHFR* Glu429Ala polymorphism with OS in these two cohorts is detected under different genetic models.

### Explorative Analyses for Overall Survival and the *MTHFR* Glu429Ala Polymorphism

While this interesting association pattern of the *MTHFR* Glu429Ala polymorphism with OS in two cohorts may also be explained by the reduced statistical power to detect the effects of each genotype groups, we also performed additional explorative analyses to investigate other possibilities. First, to test whether the association of different genotypes could be due to relatively higher median age in the validation cohort when compared to the discovery cohort (see Discussion), we performed a multivariable analysis in patients from the validation set who were under 75 years of age at the time of diagnosis (n = 149). Yet, we found that the pattern of association observed in this analysis (AC vs AA, HR: 2.02, 95% CI: [1.20–3.41], p = 0.008) was similar to that obtained in the validation cohort, suggesting age at diagnosis was not the reason for this disparity. Second, since the 5-FU efficacy might be modified by the activity of the MTHFR enzyme (see Discussion), we also tested the association of the *MTHFR* Glu429Ala genotypes with OS in patient groups stratified based on their treatment characteristics (patients treated with 5-FU based regimens versus patients not treated with it). Interestingly, in this analysis, we have found that when adjusted for age, stage, MSI status, this polymorphism was significantly associated with OS in the patients not treated with 5-FU (both CC vs AA and AC vs AA genotypes in the discovery set and AC vs AA genotypes in the validation cohort), but not in the patients treated with 5-FU based regimens (**Tables 5** and **6**). Analysis of the interaction between the *MTHFR* Glu429Ala polymorphism and the 5-FU treatment status in both the discovery and the validation cohorts did not reveal a statistically significant interaction between these two variables.

**Table 5:** The *MTHFR* Glu429Ala polymorphism and overall survival in the discovery cohort patients (stratified by treatment with 5-Fluorouracil).

	Treated with 5-FU			Not treated with 5-FU		
	Discovery cohort (n = 310)			Discovery cohort (n = 191)		
#Variable	HR (95% CI)	p-value	n	HR (95% CI)	p-value	n
***MTHFR* Glu429Ala**		0.206			**0.005**	
AC vs AA	0.88 (0.61–1.28)	0.514	143 vs 140	**2.47 (1.15–5.27)**	**0.020**	85 vs 92
CC vs AA	1.51 (0.85–2.68)	0.161	27 vs 140	**6.08 (1.95–18.93)**	**0.002**	14 vs 92
**Age**	1.02 (1.00–1.04)	0.132		**1.09 (1.04–1.14)**	**0.001**	
**Stage**		**<0.001**			**<0.001**	
II vs I	2.56 (0.35–18.81)	0.356	104 vs 7	0.67 (0.31–1.41)	0.289	89 vs 87
III vs I	2.58 (0.36–18.76)	0.349	154 vs 7	**7.70 (2.76–21.48)**	**<0.001**	10 vs 87
IV vs I	**15.84 (2.16–115.98)**	**0.007**	45 vs 7	2.98 (0.83–10.66)	0.093	5 vs 87
**MSI-H vs MSI-L/MSS**	**0.08 (0.01–0.57)**	**0.012**	23 vs 287	0.53 (0.16–1.82)	0.315	32 vs 159

5-FU: 5-fluorouracil, CI: confidence interval, HR: hazard ratio, n: number of patients, vs: versus.

#The referent categories are underlined. Please note that reflecting the small numbers of patients, the CIs for the effect estimate in stage IV patients are quite wide and should not be interpreted as an accurate estimation.

**Table 6:** The *MTHFR* Glu429Ala polymorphism and overall survival in the validation cohort patients (stratified by treatment with 5-Fluorouracil).

	Treated with 5-FU			Not treated with 5-FU		
	Validation cohort (n = 87)			Validation cohort (n = 141)		
#Variable	HR (95% CI)	p-value	n	HR (95% CI)	p-value	n
***MTHFR* Glu429Ala**		0.676			**0.032**	
AC vs AA	1.34 (0.70–2.56)	0.382	41 vs 39	**1.70 (1.10–2.63)**	**0.017**	57 vs 71
CC vs AA	1.07 (0.35–3.29)	0.903	7 vs 39	0.82 (0.36–1.88)	0.637	13 vs 71
**Age**	1.02 (0.99–1.05)	0.189		**1.06 (1.03–1.08)**	**<0.001**	
**Stage**		**<0.001**			**<0.001**	
II vs I	0.54 (0.14–2.10)	0.372	26 vs 6	1.36 (0.73–2.54)	0.331	56 vs 36
III vs I	1.42 (0.42–4.78)	0.570	42 vs 6	**3.61 (1.83–7.12)**	**<0.001**	23 vs 36
IV vs I	**6.32 (1.72–23.16)**	**0.005**	13 vs 6	**11.32 (5.52–23.20)**	**<0.001**	26 vs 36
**MSI-H vs MSI-L/MSS**	0.34 (0.08–1.46)	0.146	8 vs 79	**0.37 (0.15–0.94)**	**0.037**	13 vs 128

5-FU: 5-fluorouracil, CI: confidence interval, HR: hazard ratio, n: number of patients, vs: versus.

#The referent categories are underlined. Please note that reflecting the small numbers of patients, the CIs for the effect estimate in stage IV patients are quite wide and should not be interpreted as an accurate estimation.

### Disease Free Survival Analysis in the Validation Cohort

In the multivariable analysis of DFS in the validation cohort, the association of the *ERCC5* His46His polymorphism with DFS was also detected as follows: compared to those patients with the CC genotype, patients with TT and the CT genotypes had shorter DFS (HR: 1.81, 95% CI: [1.11–2.94], p = 0.018 and HR: 1.48, 95%CI: [1.02–2.17], p = 0.041), respectively (**Table 4**).

## Discussion

The main result of our study is that the associations of the *MTHFR* Glu429Ala polymorphism with the overall survival and the *ERCC5* His46His polymorphism with the disease-free survival were detected in two separate cohorts of colorectal cancer patients.

Two other studies have also reported the *MTHFR* Glu429Ala polymorphism to be associated with OS in colorectal cancer patients. In one study conducted in a Spanish cohort [23], patients carrying the C allele (AC+CC genotype) had worse survival than those with AA genotype, when adjusted for clinicopathological variables. Similarly, in another study [24] performed in metastatic colon cancer patients, female patients with AC+CC genotypes for the *MTHFR* Glu429Ala had worse OS than female patients with AA genotype in univariate analysis. However, in six other studies, no association was observed between the *MTHFR* Glu429Ala polymorphism and OS in colorectal cancer in univariate or multivariable analyses [25]–[30].

The role of the MTHFR enzyme and its Glu429Ala polymorphism in colorectal cancer prognosis is not well-known. However, MTHFR has been biologically investigated in great detail (i.e. its role in folate metabolism and 5-FU mechanism of action). In addition, the Glu429Ala polymorphism has been previously shown to cause moderate reduction in the activity of MTHFR enzyme; the Ala/Ala homozygotes have close to 60% of the normal MTHFR activity and the heterozygotes have ∼80% of the normal MTHFR activity [31], [32].

**Figure 2:**
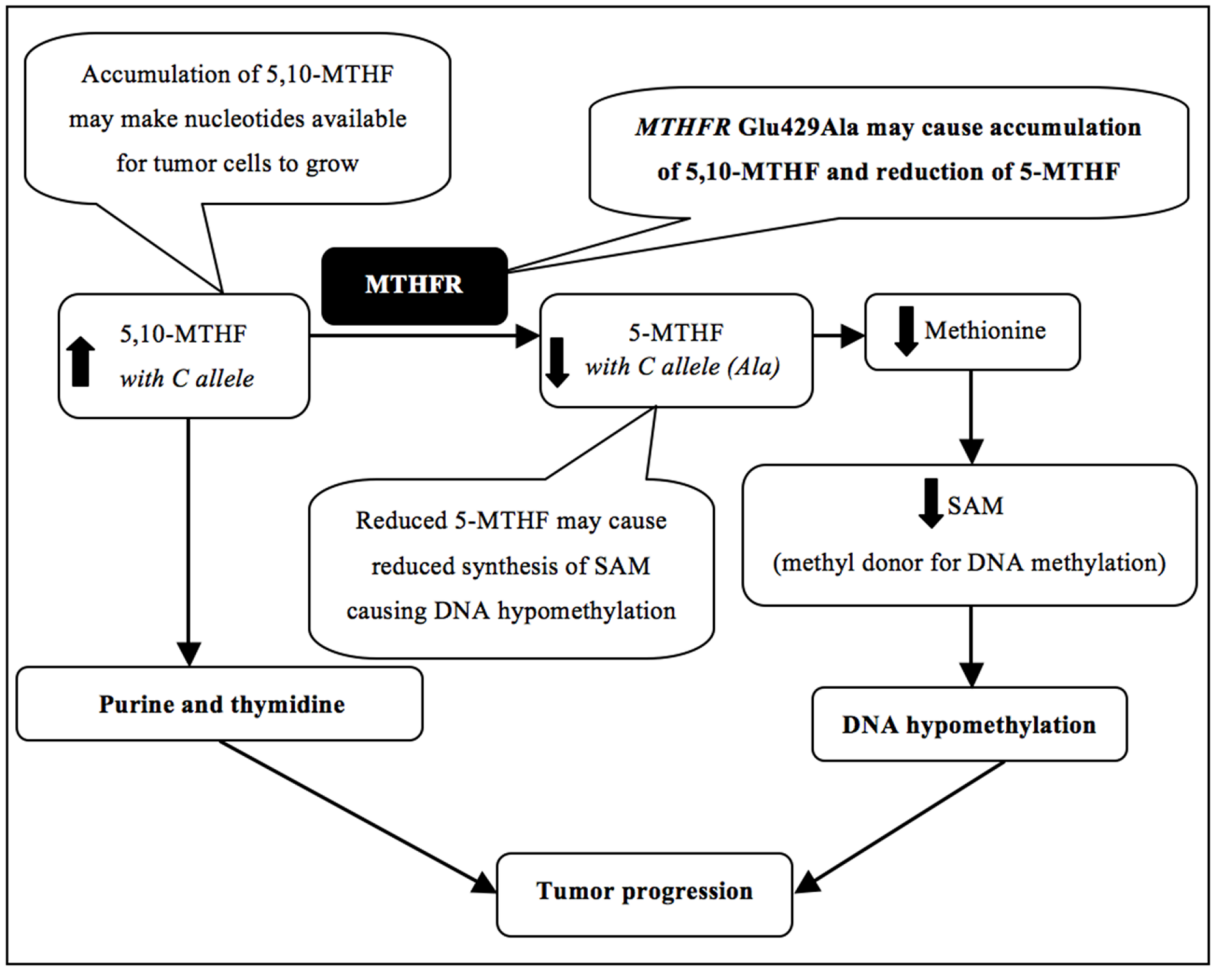
5,10-MTHF: 5,10-methylene tetrahydrofolate, 5-MTHF: 5-methyl tetrahydrofolate, MTHFR: methylene tetrahydrofolate reductase, SAM: S-adenosyl methionine. Arrows indicate the potential consequences of the polymorphism on biological processes depicted.

In folate metabolism, one of the biological activities of the MTHFR enzyme is to convert the 5,10-methylenetetrahydrofolate (5,10-MTHF) to 5-methyltetrahydrofolate (5-MTHF) [33]. 5,10-MTHF is predominantly used in the synthesis of purines and thymidine, the nucleotides used by the dividing cells in DNA synthesis. In addition, 5-MTHF is used in the synthesis of S-adenosyl-methionine (SAM), a key mediator in a number of methylation reactions including DNA methylation [33]. Thus reduction in MTHFR enzymatic activity due to Glu429Ala polymorphism may result in accumulation of 5,10-MTHF and concurrent reduction of 5-MTHF to a certain extent (**Figure 2**). Accumulation of 5,10-MTHF form of folate may provide increased amounts of nucleotides for DNA synthesis to the rapidly proliferating tumor cells to grow. This theory is supported by recent reports which suggest that once a colorectal adenoma has developed, folate supplementation can aid its growth and progression [34]–[37], presumably by facilitating large amounts of nucleotide precursors for tumor growth [33], [35], [36]. In another study, folate supplementation was found to be associated with progression of already developed colorectal cancer in rats [36]. Also in the Aspirin/Folate Polyp Prevention Study [34], [36], folate supplementation was associated with higher risk of advanced adenomas as well as increased number of adenomas in patients with previously developed colorectal adenomas. These findings suggest a negative effect of high folate levels in colorectal cancer prognosis. Therefore, in our cohorts, the association of the *MTHFR* Glu429Ala polymorphism with worse prognosis of colorectal cancer patients may be due to reduced activity of MTHFR and accumulation of folate which can facilitate tumor growth.

In addition, due to the inefficient MTHFR enzyme function (for example, due to the Glu429Ala polymorphism), the optimal conversion of 5,10-MTHF to 5-MTHF may also be reduced, causing reduction in the levels of 5-MTHF. This may ultimately lead to a decrease in synthesis of SAM (**Figure 2**). SAM is an important methyl donor for a large number of reactions and its deficiency can induce DNA hypomethylation. In *MTHFR* gene knockout mice, the levels of SAM as well as the extent of DNA methylation were found to be significantly reduced [38]. Thus by reducing the enzymatic activity, the *MTHFR* Glu429Ala polymorphism may lead to a similar, although a less severe consequence. In a study conducted in tumor cells from colon cancer patients, for example, DNA hypomethylation was associated with unfavorable cancer-specific survival and OS [39]. Accordingly, DNA hypomethylation due to the *MTHFR* Glu429Ala polymorphism mediated reduction in SAM levels may have also contributed to unfavorable prognosis in our cohorts.

Another finding in our study was the association of different genotypes of the *MTHFR* Glu429Ala polymorphism with OS in two separate colorectal cancer patient cohorts. To explore the potential causes of this disparity, we have focused on the differences between the discovery and validation cohorts that were known to play a biological role in MTHFR activity or the folate metabolism; namely age and treatment with 5-FU. Older individuals are known to have an impaired ability to absorb dietary folate [40] and the validation cohort in our study had a significantly higher median age compared to the discovery cohort (p<0.001). We therefore hypothesized that along with the low folate absorption in older patients, the mildly reduced MTHFR activity due to heterozygosity of *MTHFR* Glu429Ala polymorphism might have been sufficient to contribute to the unfavorable prognosis observed in the validation cohort (in this case, the Ala/Ala homozygotes would also be expected to have shorter OS; however, it was possible that this association might have been missed due to insufficient study power for comparison of CC vs AA genotypes). Therefore, we conducted a multivariable analysis in the validation cohort patients, who were <75 years of age at the time of diagnosis (similar to the patients in the discovery set). These results also showed the association of the AC genotype of the *MTHFR* Glu429Ala polymorphism with OS when compared to the AA genotype, similar to the association detected in the entire validation cohort. Therefore, it is not likely that increased age together with the *MTHFR* Ala429 variant and their effect on folate mechanism may explain the association of two different genotypes in our discovery and validation cohorts.

We then focused on the 5-FU and its effect on the folate metabolism. 5-FU is routinely used in the colorectal cancer chemotherapy and one of its anti-neoplastic mechanisms is the inhibition of thymidine synthesis. In the process of thymidine synthesis inhibition, 5,10-MTHF stabilizes the chemical complex necessary for inhibition of thymidylate synthase (TYMS) enzyme [41]. In the presence of increased concentration of 5,10-MTHF, the inhibition of thymidine synthesis and thus the efficacy of 5-FU is expected to increase and this has been demonstrated *in vitro* in human colon cancer cells [42]. However, in multiple prognostic studies, statistical association of the *MTHFR* Glu429Ala polymorphism with response to treatment with 5-FU based chemotherapy in colorectal cancer patients was not detected [25], [26], [43]–[49] suggesting that *MTHFR* Glu429Ala polymorphism may not affect the efficacy of 5-FU based treatments. In the present study too, no significant association of *MTHFR* Glu429Ala was found in patients treated with 5-FU based chemotherapy (although we cannot fully rule out the possibility of insufficient study power to detect an effect). However, in our study, *MTHFR* Glu429Ala polymorphism was associated with shorter OS in patients, who were not treated with 5-FU in both the discovery and the validation cohorts (**Tables 5** and **6**). On further analyses, we found that the majority of non-5-FU treated patients were stage I and II (92%) and had colon tumors (81.4%), who generally receive surgical treatment without 5-FU-based chemotherapy. Therefore, these results suggest that the reduced MTHFR activity due to Glu429Ala polymorphism may be associated with shorter OS, and thus may be a promising adverse prognostic marker, in early stage colon cancer patients or those patients not treated with 5-FU. Alternatively, other polymorphisms highly linked with *MTHFR* Glu429Ala polymorphism may be the reason for this association (**Methods S2** and **Figure S1** in **File S1**). While these results are needed to be confirmed with further studies, to our knowledge, this is the first study that identified a potential prognostic significance of the *MTHFR* Glu429Ala polymorphism in colorectal cancer patients not treated with 5-FU.

In the present study, we also show that the TT genotype of the *ERCC5* His46His polymorphism is associated with shorter DFS in the two colorectal cancer patient cohorts investigated (**Table 4**). To our knowledge, this is the first study that reports the association of the *ERCC5* His46His polymorphism with DFS in colorectal cancer patients. ERCC5 is one of the endonucleases functioning in the nucleotide excision repair. *ERCC5* His46His is a synonymous and non-splice site polymorphism and its impact on function of ERCC5 protein is uncertain. Previously, the TT genotype of this polymorphism was reported to be associated with short progression free survival (PFS) in advanced colorectal cancer patients receiving oxaliplatin [49] and short PFS and OS times in stage I and II head and neck cancer patients receiving radiotherapy [50]. Radiotherapy-resistant lung cancer cells have also shown an up-regulation of *ERCC5* [51]. Additionally, in a study of ovarian cancer patients treated with platinum-based chemotherapeutic drugs, loss of heterozygosity (LOH) of *ERCC5* and down regulation of this gene were associated with a favorable PFS, presumably due to increased efficacy of these drugs [52]. Whether His46His polymorphism causes up-regulation or down-regulation of *ERCC5* is presently unknown and functional characterization of the polymorphism is required to understand its potential prognostic role in colorectal cancer. Alternatively, since this synonymous polymorphism does not alter the amino acid sequence in the protein, it is also likely that rather than the polymorphism itself, another highly correlated polymorphism in the same LD block may have a biological impact on disease progression and survival (**Methods S2** and **Figure S2** in **File S1**).

Twenty-four of the 26 selected polymorphisms (excluding one polymorphism which was not included into the statistical analysis in this study due to its low minor allele frequency) did not show an association with survival in our discovery cohort and previously reported associations were thus not replicated in colorectal cancer patients from Newfoundland. Such a lack of concordance in results of genetic prognostic studies is a common observance due to significant heterogeneity in the cohort characteristics and study design amongst different studies. For example, variations in patient ethnicities, treatment characteristics, follow-up times and clinical characteristics are regarded as critical reasons for the discordance in results of genetic prognostic studies in different study cohorts [53], [54]. In addition, the discovery and validation cohorts used in this study are predominantly composed of Caucasian patients, with follow-up times of up to 10 years and other clinicopathological features described in **Table 2**. These features may not be shared by the other published cohorts (**Table S1** in **File S1**), which may have contributed to differences in the results. Finally, there are differences between the discovery and validation cohorts in terms of several demographic and clinicopathological features (sex, age, stage, grade and invasion status, 5-FU treatment status) and OS and DFS follow up times (**Table 2**). These differences as well as the small sample size of the validation cohort may also explain why no associations of *ERCC5* His46His, *SERPINE1 −*675indelG and *GSTM1* gene deletion polymorphisms with OS were detected in the validation cohort. Therefore, it is possible that associations of *ERCC5* His46His, *SERPINE1 −*675indelG and *GSTM1* gene deletion polymorphisms with OS may be detected in other cohorts with similar characteristics to the discovery cohort. Alternatively, the associations detected in the discovery cohort could be false-positive associations.

The limitations of this study are the dissimilarities between the discovery and validation cohorts, the fact that the discovery cohort was biased towards early stage patients, small size of the validation cohort, the short follow-up time in the validation cohort, especially for DFS, the limited number of genes and polymorphisms investigated, and the limited gene coverage (i.e. other polymorphisms in these genes were not studied). The main strength of this study is the relatively large sample size of the discovery cohort. This is also one of the few studies in colorectal cancer that attempted to replicate results in an additional patient cohort.

## Supporting Information

File S1 **Supporting information.** Figure S1 The circled SNP is rs1801131 (*MTHFR* Glu429Ala), which lies in a 12kb LD block. The black squares indicate other highly correlated SNPs (r^2^>0.80). Figure S2 The circled SNP is rs1046678 (*ERCC5* His46His), which lies in a 23kb LD block. The black squares indicate other highly correlated SNPs (r^2^>0.80). Table S1 n: number. Table S2 ^*^Assay ID by Applied Biosystems (CA, USA). Underlined are the sequences on probes that are complementary to alleles they recognize. Assays for rs1799750 in *MMP1* gene and rs1799889 in *SERPINE1* gene were custom designed. Assays for rs1801131 in *MTHFR* gene and rs1047768 in *ERCC5* gene were predesigned by Applied Biosystems. Primer and probe sequences for these assays are not available since they are proprietary of Applied Biosystems. Seq: sequence. Table S3 n/a: not applicable. Polymorphisms with χ2 value greater than 3.84 were considered to be deviating from HWE (p<0.05). *For these gene deletions, since heterozygote genotype cannot be determined by the genotyping method applied, HWE was not calculated. All polymorphisms were investigated in this study regardless of their deviations from the HWE as these deviations may also be attributed to the fact that the Newfoundland population is considered a genetically isolated population [49]. Nevertheless, it is worth noting that while the *OGG1* Ser326Cys polymorphism that deviated from the HWE was included in the DFS multivariable model of the discovery cohort, its genotype data was not available for the validation cohort patients. Thus, the main conclusion on the disease-free survival analysis that the *ERCC5* His46His polymorphism was associated with DFS in both the discovery and the validation patient cohorts is not affected by including this polymorphism in the DFS analysis of the discovery cohort. Table S4 CI: confidence interval, HR: hazard ratio, MSI: microsatellite instability, n: number of patients, vs: versus. Table S5 CI: confidence interval, HR: hazard ratio, MSI: microsatellite instability, n: number of patients, vs: versus. Table S6 CI: confidence interval, HR: hazard ratio, MSI: microsatellite instability, n: number of patients, vs: versus. Table S7 CI: confidence interval, HR: hazard ratio, MSI: microsatellite instability, n: number of patients, vs: versus. Methods S1 Genotyping reactions. Methods S2 Construction of linkage disequilibrium (LD) maps.
(DOC)
