# Supplementary material for: MTHFR Glu429Ala and ERCC5 His46His Polymorphisms Are Associated with Prognosis in Colorectal Cancer Patients: Analysis of Two Independent Cohorts from Newfoundland
Source: PLoS One. 2013 Apr 23;8(4):e61469. doi: 10.1371/journal.pone.0061469 (PMC3634085; doi:10.1371/journal.pone.0061469)
Supplement: File S1 — Supporting information. Figure S1 The circled SNP is rs1801131 (MTHFR Glu429Ala), which lies in a 12kb LD block. The black squares indicate other highly correlated SNPs (r2>0.80). Figure S2 The circled SNP is rs1046678 (ERCC5 His46His), which lies in a 23kb LD block. The black squares indicate other highly correlated SNPs (r2>0.80). Table S1 n: number. Table S2 *Assay ID by Applied Biosystems (CA, USA). Underlined are the sequences on probes that are complementary to alleles they recognize. Assays for rs1799750 in MMP1 gene and rs1799889 in SERPINE1 gene were custom designed. Assays for rs1801131 in MTHFR gene and rs1047768 in ERCC5 gene were predesigned by Applied Biosystems. Primer and probe sequences for these assays are not available since they are proprietary of Applied Biosystems. Seq: sequence. Table S3 n/a: not applicable. Polymorphisms with χ2 value greater than 3.84 were considered to be deviating from HWE (p<0.05). *For these gene deletions, since heterozygote genotype cannot be determined by the genotyping method applied, HWE was not calculated. All polymorphisms were investigated in this study regardless of their deviations from the HWE as these deviations may also be attributed to the fact that the Newfoundland population is considered a genetically isolated population [49]. Nevertheless, it is worth noting that while the OGG1 Ser326Cys polymorphism that deviated from the HWE was included in the DFS multivariable model of the discovery cohort, its genotype data was not available for the validation cohort patients. Thus, the main conclusion on the disease-free survival analysis that the ERCC5 His46His polymorphism was associated with DFS in both the discovery and the validation patient cohorts is not affected by including this polymorphism in the DFS analysis of the discovery cohort. Table S4 CI: confidence interval, HR: hazard ratio, MSI: microsatellite instability, n: number of patients, vs: versus. Table S5 CI: confidence interval, HR: hazard ratio, [file pone.0061469.s001.doc]

**Supplementary Information**

**Table S1.** Polymorphisms included into the present study that were reported to be associated with overall survival in colorectal cancer patients in at least one previously published study.

| **Gene symbol** | **Polymorphism** | **rs number** | **Ethnicity** | **Sample size** | **Reference** | **Included in this study** |
| --- | --- | --- | --- | --- | --- | --- |
| *CCND1* | Pro241Pro A/G (NM_053056.2:c.723G>A) | rs9344 | Mixed population | n=39 (Caucasian=31, Asian=6, Hispanic=2) | [1] | yes |
| *DCC* | Arg201Gly C/G (NP_005206.2:p.Arg201Gly) | rs2229080 | Caucasian | n=59 | [2] | yes |
| *EGF* | A61G in 5'-UTR (NM_001963.3:c.-382A>G) | rs4444903 | Caucasian | n=110 | [3] | failed to be genotyped in MassArray^(R)^ reaction |
| *EGFR* | (CA)n repeat in intron 1 | n/a | Caucasian | n=110 | [3] | genotyping method not available in our lab |
| *EGFR* | Arg497Lys G/A (Arg521Lys) (NP_005219.2:p.Arg521Lys) | rs2227983, rs11543848 | Caucasian | n=32 | [4] | yes |
| *EGFR* | Arg497Lys G/A (Arg521Lys) (NP_005219.2:p.Arg521Lys) | rs2227983, rs11543848 | Mixed population | n=318 (Asian=43, Black=15, Hispanic=24, Native American=2, White=234) | [5] | yes |
| *EGFR* | Arg497Lys G/A (Arg521Lys) (NP_005219.2:p.Arg521Lys) | rs2227983, rs11543848 | Asian | n=209 | [6] | yes |
| *ERCC1* | Asn118Asn C/T (NM_001166049.1:c.354T>C) | rs11615 | Asian | n=166 | [7] | yes |
| *ERCC1* | Asn118Asn C/T (NM_001166049.1:c.354T>C) | rs11615 | Asian | n=168 | [8] |  |
| *ERCC1* | Asn118Asn C/T (NM_001166049.1:c.354T>C) | rs11615 | Caucasian | n=377 | [9] | yes |
| *ERCC1* | Asn118Asn C/T (NM_001166049.1:c.354T>C) | rs11615 | Mixed population | n=106 (White=75, Hispanic=14, Black=6, Asian=11) | [10] | yes |
| *ERCC1* | Asn118Asn C/T (NM_001166049.1:c.354T>C) | rs11615 | Mixed population | n=106 (Caucasian=75, Hispanic=14, Black=6, Asian=11) | [11] | yes |
| *ERCC1* | Asn118Asn C/T (NM_001166049.1:c.354T>C) | rs11615 | Asian | n=113 | [12] | yes |
| *ERCC2* | Lys751Gln G/T (NP_000391.1:p.Lys751Gln) | rs13181 | Asian | n=166 | [7] | yes |
| *ERCC2* | Lys751Gln G/T (NP_000391.1:p.Lys751Gln) | rs13181 | Mixed population | n=73 (Caucasian=55, Hispanic=11, Black=2, Asian=5) | [13] | yes |
| *ERCC2* | Lys751Gln G/T (NP_000391.1:p.Lys751Gln) | rs13181 | Mixed population | n=106 (Caucasian=75, Hispanic=14, Black=6, Asian=11) | [11] | yes |
| *ERCC2* | Lys751Gln G/T (NP_000391.1:p.Lys751Gln) | rs13181 | Asian | n=217 | [14] | yes |
| *ERCC2* | Lys751Gln G/T (NP_000391.1:p.Lys751Gln) | rs13181 | Caucasian | n=39 | [15] | yes |
| *ERCC5* | His46His C/T (NM_000123.2:c.138T>C) | rs1047768 | Caucasian | n=42 | [16] | yes |
| *EXO1* | Pro757Leu C/T (NP_003677.3:p.Pro757Leu) | rs9350 | Asian | n=114 | [17] | yes |
| *FAS* | -670A/G in promoter (NM_001141945.1:c.-24+733T>C) | rs1800682 | Caucasian | n=384 | [18] | yes |
| *FGFR4* | Gly388Arg A/G (NP_002002.3:p.Gly388Arg) | rs351855 | Caucasian | n=82 | [19] | yes |
| *GSTM1* | gene deletion | n/a | Caucasian | n=102 | [20] | yes |
| *GSTP1* | Ile105Val A/G (NP_000843.1:p.Ile105Val) | rs1695 | Mixed population | n=107 (Caucasian=77, Hispanic=14, Black=5, Asian=11) | [21] | yes |
| *GSTP1* | Ile105Val A/G (NP_000843.1:p.Ile105Val) | rs1695 | Mixed population | n=106 (Caucasian=75, Hispanic=14, Black=6, Asian=11) | [11] | yes |
| *GSTP1* | Ile105Val A/G (NP_000843.1:p.Ile105Val) | rs1695 | Caucasian | n=125 | [22] | yes |
| *GSTP1* | Ile105Val A/G (NP_000843.1:p.Ile105Val) | rs1695 | Asian | n=122 | [23] | yes |
| *GSTP1* | Ile105Val A/G (NP_000843.1:p.Ile105Val) | rs1695 | Asian | n=166 | [7] | yes |
| *GSTP1* | Ile105Val A/G (NP_000843.1:p.Ile105Val) | rs1695 | Caucasian | n=60 | [24] | yes |
| *GSTT1* | gene deletion | n/a | Caucasian | n=361 | [25] | yes |
| *IL6* | -174G/C in promoter (NG_011640.1:g.4880C>G) | rs1800795 | Caucasian | n=308 | [26] | yes |
| *MLH1* | Ile219Val A/G (NP_000240.1:p.Ile219Leu) | rs1799977 | Caucasian | n=140 | [27] | yes |
| *MMP1* | -1607 indelG in promoter (NG_011740.1:g.3471delG) | rs1799750 | Caucasian | n=503 | [28] | yes |
| *MMP1* | -1607 indelG in promoter (NG_011740.1:g.3471delG) | rs1799750 | Caucasian | n=201 | [29] | yes |
| *MMP2* | -1306C/T in promoter (NG_008989.1:g.3726C>T) | rs243865 | Caucasian | n=215 | [30] | yes |
| *MTHFR* | Ala222Val C/T (NP_005948.3:p.Ala222Val) | rs1801133 | Caucasian | n=544 | [31] | yes |
| *MTHFR* | Ala222Val C/T (NP_005948.3:p.Ala222Val) | rs1801133 | Caucasian | n=157 | [32] | yes |
| *MTHFR* | Glu429Ala A/C (NP_005948.3:p.Glu429Ala) | rs1801131 | Mixed population | n=318 (White=234, Black=15, Asian=43, Hispanic=24, Native American=2) | [33] | yes |
| *MTHFR* | Glu429Ala A/C (NP_005948.3:p.Glu429Ala) | rs1801131 | Caucasian | n=143 | [34] | yes |
| *OGG1* | Ser326Cys C/G (NP_002533.1:p.Ser326Cys) | rs1052133 | Caucasian | n=91 | [35] | yes |
| *PTGS2* | -765G/C in promoter (NT_004487.19:g.38138963C>G) | rs20417 | Caucasian | n=195 | [36] | failed to be genotyped in MassArray^(R)^ reactions |
| *PTGS2* | c.3618A/G in 3'-UTR (NM_000963.2:c.*1803A>G) | rs4648298 | Caucasian | n=284 | [37] | yes |
| *SERPINE1* | -675indel4G/5G in promoter (NG_013213.1:g.4332_4333insA) | rs1799889 | Caucasian | n=308 | [38] | yes |
| *TP53* | Arg72Pro C/G (NP_000537.3:p.Pro72Arg) | rs1042522 | Caucasian | n=102 | [20] | failed to be genotyped in MassArray^(R)^ reactions |
| *TYMS* | 2/3 repeats of 28bp in 5'-UTR | rs34743033 | Caucasian | n=166 | [39] | yes |
| *TYMS* | 2/3 repeats of 28bp in 5'-UTR | rs34743033 | Asian | n=121 | [40] | yes |
| *TYMS* | indel6bp in 3'-UTR | rs16430 | Caucasian | n=129 | [41] | yes |
| *TYMS* | indel6bp in 3'-UTR | rs16430 | Caucasian | n=90 | [42] | yes |
| *TYMS* | indel6bp in 3'-UTR | rs16430 | Mixed population | n=520 (White=450, Black=36, Hispanic=16, Asian=9, Other=9) | [43] | yes |
| *VEGFA* | -634G/C in 5'-UTR (NM_001025366.1:c.-94C>G) | rs2010963 | Caucasian | n=312 | [44] | yes |
| *VEGFA* | +936C/T in 3'-UTR (NM_001025366.1:c.*237C>T) | rs3025039 | Caucasian | n=312 | [44] | yes |
| *XRCC1* | Arg399Gln G/A (NP_006288.2:p.Gln399Arg) | rs25487 | Caucasian | n=91 | [35] | yes |
| *XRCC1* | Arg399Gln G/A (NP_006288.2:p.Gln399Arg) | rs25487 | Caucasian | n=38 | [15] | yes |
| *XRCC1* | Arg399Gln G/A (NP_006288.2:p.Gln399Arg) | rs25487 | Asian | n=113 | [12] | yes |
| *XRCC1* | Arg399Gln G/A (NP_006288.2:p.Gln399Arg) | rs25487 | Caucasian | n=377 | [9] | yes |
| *XRCC1* | Arg399Gln G/A (NP_006288.2:p.Gln399Arg) | rs25487 | Asian | n=54 | [45] | yes |
| *XRCC3* | Thr241Met C/T (NP_001093588.1:p.Thr241Met) | rs861539 | Caucasian | n=377 | [9] | yes |

**Methods S1. Genotyping reactions**

**a) Sequenom MassArray® genotyping:** In the discovery cohort, 22 polymorphisms were genotyped using the Sequenom MassArray® system at an outsourced genomics facility (Analytical Genetics Technology Centre facility at the University Health Network, Toronto).

**b) TaqMan® SNP genotyping assays:** The *MMP1* -1607indelG and *SERPINE1* -675indelG polymorphisms in the discovery cohort and the *MTHFR* Glu429Ala*, SERPINE1* -675indelG and *ERCC5* His46His polymorphisms in the validation cohort were genotyped using the TaqMan® SNP genotyping assays (**Supplementary Table S2**). For these reactions, the primers and fluorescent probes were either custom designed using the Custom TaqMan® Assay Design Tool [46], or pre-designed assays were ordered from the Applied Biosystems (CA, USA). For each sample, a 10µl reaction mix contained 1µl DNA solution (3-5ng/µl), 5µl 2X TaqMan® Universal PCR master mix (Roche, NJ, USA), 0.25µl 20X TaqMan® assay mix (Applied Biosystems, CA, USA) and sterile water added up to the final reaction volume of 10µl. The reaction conditions were: 50ºC for 2' (activation of AmpErase® UNG in TaqMan® Universal PCR master mix) and 95ºC for 10' (activation of AmpliTaq Gold® DNA polymerase in TaqMan® Universal PCR master mix); followed by 40 cycles of 95ºC for 15" (DNA denaturation) and 60ºC for 1' (annealing and extension of primers). Reactions were run on a 7900HT Fast Real-Time PCR System (Applied Biosystems, CA, USA). Genotypes were called by the SDS 2.4 software accompanying the 7900HT Fast Real-Time PCR System. The genotype plots were also manually examined to finalize the genotypes.

**c) Gel electrophoresis of PCR amplified products**: Deletions of the *GSTT1* and *GSTM1* genes in both the discovery and the validation cohorts were determined using a multiplex PCR reaction method described by Arand et al [47]. For each sample, the multiplex reaction mix contained 1µl DNA solution (3-5ng/µl), 5µl 2X AmpliTaq Gold® 360 Master Mix (Applied Biosystems, CA, USA), 1µl primer mix containing 10µM each of forward and reverse primer for amplification of *GSTT1, GSTM1* and *ALB* (positive control) gene fragments, 0.25µl GC enhancer (Applied Biosystems, CA, USA) with sterile water added up to the final reaction volume (10µl). The PCR conditions were 95ºC for 10' followed by 35 cycles of 95ºC for 30", 64ºC for 30", and 72ºC for 1', followed by a final elongation step at 72ºC for 7'. PCRs were performed in either a 7900HT Fast Real-Time PCR System (Applied Biosystems, CA, USA) or a Veriti 96-well fast thermal cycler (Applied Biosystems, CA, USA). The PCR products were run on 1.5% agarose gels containing SYBR® Safe DNA gel stain (Invitrogen, Oregon, USA) at 70 volts. Gel images were taken under ultraviolet (UV) transillumination. Genotypes were determined manually by inspection of gel images.

The variable number of tandem repeat (VNTR) polymorphism in the untranslated region (UTR) of the *TYMS* gene is usually present as two or three tandem repeats (2R/3R) of a 28 base pair (bp) DNA sequence (rs34743033). This VNTR was genotyped using gel electrophoresis of PCR amplified products following a method previously described by Carlini et al [48]. In brief, the PCR reaction mix contained 1µl DNA solution (3-5ng/µl), 5µl 2X AmpliTaq Gold® 360 Master Mix (Applied Biosystems, CA, USA), 2µl primer mix containing 10µM forward and reverse primer, 0.5µl GC enhancer (Applied Biosystems, CA, USA), and sterile water added up to the final reaction volume (10µl). The reaction conditions for this PCR amplification were the same as described for GSTM1/GSTT1 genotyping, with the differences of 34 reaction cycles and primer annealing temperature of 70ºC. The PCR products were run on 4% agarose gels, DNA bands were visualized and genotypes were determined as described above.

**Table S2. Primer, probe and assay ID information for polymorphisms genotyped by the TaqMan® SNP genotyping assays**

| **SNP** | **rs1799750 (*MMP1*)** | **rs1799889 (*SERPINE1*)** | **rs1801131 (*MTHFR*)** | **rs1047768 (*ERCC5*)** |
| --- | --- | --- | --- | --- |
| ***Assay ID** | **AHVI4S6** | **AHWR2ZE** | **C_850486_20** | **C_1891769_20** |
| **Forward primer Seq.** | ACATGTTATGCCACTTAGATGAGGAAA | AGACAAGGTTGTTGACACAAGAGA |  |  |
| **Reverse primer Seq.** | CGTCAAGACTGATATCTTACTCATAAACAATACTTC | GGCCGCCTCCGATGATAC | not available | not available |
| ****Probe 1 Seq.** | TGAGATAAGTCATATCCTTTC | ACGGCTGACTCCCCCAC |  |  |
| *****Probe 2 Seq.** | TGAGATAAGTCATATCTTTC | CGGCTGACTCCCCAC |  |  |

**Table S3. The Hardy-Weinberg Equilibrium (HWE) calculations for the polymorphisms investigated in this study.**

| **Gene** | **Polymorphism** | **SNP ID** | **χ^2^ value** | **p ≤ 0.05** | **Genotypes in HWE** |
| --- | --- | --- | --- | --- | --- |
| **Discovery cohort** | | | | | |
| *CCND1* | Pro241Pro, A/G | rs9344 | 0.01 | no | yes |
| *DCC* | Arg201Gly, C/G | rs2229080 | 0.7 | no | yes |
| *EGFR* | Arg521Lys, G/A | rs2227983 | 2.61 | no | yes |
| *FGFR4* | Gly388Arg, A/G | rs351855 | 2.68 | no | yes |
| *ERCC1* | Asn118Asn, C/T | rs11615 | 3.46 | no | yes |
| *ERCC2* | Lys751Gln, G/T | rs13181 | 4.6 | yes | no |
| *ERCC5* | His46His, C/T | rs1047768 | 0.6 | no | yes |
| *EXO1* | Pro757Leu, C/T | rs9350 | 0.01 | no | yes |
| *OGG1* | Ser326Cys, C/G | rs1052133 | 4.32 | yes | no |
| *MLH1* | Ile219Val, A/G | rs1799977 | 0.1 | no | yes |
| *XRCC1* | Arg399Gln, G/A | rs25487 | 0.05 | no | yes |
| *XRCC3* | Thr241Met, C/T | rs861539 | 5.42 | yes | no |
| *FAS* | c.-24+733T>C | rs1800682 | 0.81 | no | yes |
| *GSTM1* | *gene deletion | na | n/a | n/a | n/a |
| *GSTP1* | Ile105Val, A/G | rs1695 | 0.01 | no | yes |
| *GSTT1* | *gene deletion | na | n/a | n/a | n/a |
| *IL6* | -174G/C in promoter | rs1800795 | 0.1 | no | yes |
| *PTGS2* | c.3618A/G in 3’-UTR | rs4648298 | 0.14 | no | yes |
| *MMP1* | -1607 indel G in promoter | rs1799750 | 0.76 | no | yes |
| *MMP2* | -1306C/T in promoter | rs243865 | 2.07 | no | yes |
| *MTHFR* | Ala222Val, C/T | rs1801133 | 0.15 | no | yes |
| *MTHFR* | Glu429Ala, A/C | rs1801131 | 1.66 | no | yes |
| *TYMS* | 2R/3R in 5’-UTR | rs34743033 | 1.28 | no | yes |
| *TYMS* | indel 6 bp in 3’-UTR | rs16430 | 0.02 | no | yes |
| *SERPINE1* | -675 indelG in promoter | rs1799889 | 1.12 | no | yes |
| *VEGFA* | -634G/C in 5’-UTR | rs2010963 | 9.58 | yes | no |
| *VEGFA* | +936C/T in 3’-UTR | rs3025039 | 0.5 | no | yes |
| **Validation cohort** | | | | | |
| *MTHFR* | Glu429Ala A/C | rs1801131 | 0.02 | no | yes |
| *ERCC5* | His46His C/T | rs1047768 | 0.28 | no | yes |
| *SERPINE1* | -675 indelG in promoter | rs1799889 | 1.62 | no | yes |
| *GSTM1* | ***gene deletion | n/a | n/a | n/a | n/a |

**Table S4. Multivariable analysis results for the *MTHFR* Glu429Ala polymorphism in the discovery cohort, the dominant genetic model**

|  | **Discovery cohort (n=504)** | | | | |
| --- | --- | --- | --- | --- | --- |
| **Variable** | **p-value** | **HR (95% CI)** | | | **n** |
| *MTHFR* rs1801131 (CA+CC vs AA) | .277 | 1.186 (0.872-1.612) | | | 272 vs 232 |
| Sex (males vs females) | .108 | 1.312 (0.942-1.827) | | | 313 vs 191 |
| Age at diagnosis | **.034** | **1.019 (1.001-1.036)** | | |  |
| Stage | **<0.001** |  |  |  |  |
| Stage (II vs I) | .121 | 1.560 (0.890-2.734) | | | 194 vs 95 |
| Stage (III vs I) | **.003** | **2.304 (1.323-4.011)** | | | 165 vs 95 |
| Stage (IV vs I) | **<0.001** | **11.094 (6.160-19.979)** | | | 50 vs 95 |
| MSI Status (MSI-H vs MSI-L/MSS) | **.002** | **0.201 (0.074-0.547)** | | | 56 vs 448 |

CI: confidence interval, HR: hazard ratio, MSI: microsatellite instability, n: number of patients, vs: versus.

**Table S5. Multivariable analysis results for the *MTHFR* Glu429Ala polymorphism in the discovery cohort, the recessive genetic model**

|  | **Discovery cohort (n=504)** | | |
| --- | --- | --- | --- |
| **Variable** | **p-value** | **HR (95% CI)** | **n** |
| *MTHFR* rs1801131 (CC vs CA+AA) | **.014** | **1.797 (1.129-2.861)** | 42 vs 462 |
| Sex (male vs female) | .123 | 1.296 (0.932-1.802) | 313 vs 191 |
| Age at diagnosis | .056 | 1.017 (1.000-1.034) |  |
| Stage | **<0.001** |  |  |
| II vs I | .173 | 1.481 (0.842-2.603) | 194 vs 95 |
| III vs I | **.006** | **2.191 (1.257-3.820)** | 165 vs 95 |
| IV vs I | **<0.001** | **10.956 (6.082-19.736)** | 50 vs 95 |
| MSI Status (MSI-H vs MSS/MSI-L) | **.002** | **0.201 (0.074-0.547)** | 56 vs 448 |

CI: confidence interval, HR: hazard ratio, MSI: microsatellite instability, n: number of patients, vs: versus.

**Table S6. Multivariable analysis results for the *MTHFR* Glu429Ala polymorphism in the validation cohort, the dominant genetic model**

|  | **Validation cohort (n=243)** | | |
| --- | --- | --- | --- |
| **Variable** | **p-value** | **HR (95% CI)** | **n** |
| *MTHFR* rs1801131 (AC+CC vs AA) | **.009** | **1.556 (1.116-2.169)** | 122 vs 121 |
| Sex (males vs females) | .216 | 1.235 (0.884-1.724) | 127 vs 116 |
| Age at diagnosis | **<0.001** | **1.049 (1.033-1.065)** |  |
| Stage | **<0.001** |  |  |
| II vs I | .694 | 1.113 (0.652-1.900) | 87 vs 48 |
| III vs I | **.001** | **2.480 (1.453-4.234)** | 67 vs 48 |
| IV vs I | **<0.001** | **9.750 (5.469-17.383)** | 41 vs 48 |
| MSI status (MSI-H vs MSI-L/MSS) | **.004** | **0.322 (0.148-0.698)** | 23 vs 220 |

CI: confidence interval, HR: hazard ratio, MSI: microsatellite instability, n: number of patients, vs: versus.

**Table S7. Multivariable analysis results for the *MTHFR* Glu429Ala polymorphism in the validation cohort, the recessive genetic model**

|  | **Validation cohort (n=243)** | | |
| --- | --- | --- | --- |
| **Variable** | **p-value** | **HR (95% CI)** | **n** |
| *MTHFR* rs1801131 (CC vs AA+AC) | .219 | 0.687 (0.377-1.251) | 21 vs 222 |
| Sex (males vs females) | .289 | 1.198 (0.858-1.674) | 127 vs 116 |
| Age at diagnosis | **<0.001** | **1.046 (1.030-1.061)** |  |
| Stage | **<0.001** |  |  |
| II vs I | .419 | 1.244 (0.733-2.112) | 87 vs 48 |
| III vs I | **<0.001** | **2.627 (1.540-4.483)** | 67 vs 48 |
| IV vs I | **<0.001** | **11.111 (6.201-19.908)** | 41 vs 48 |
| MSI status (MSI-H vs MSI-L/MSS) | **.002** | **0.289 (0.133-0.627)** | 23 vs 220 |

CI: confidence interval, HR: hazard ratio, MSI: microsatellite instability, n: number of patients, vs: versus.

**Methods S2. Construction of linkage disequilibrium (LD) maps**

Genotype data of Caucasian samples for the 100kb region containing the *MTHFR* Glu429Ala and *ERCC5* His46His polymorphisms were downloaded from the HapMap database [50]. These genotype data were then used to generate the LD maps using the Haploview 4.2 software [51] (**Supplementary Figures S1** and **S2**).

**Figure S1.** Linkage disequilibrium block containing the *MTHFR* Glu429Ala (rs1801131) polymorphism


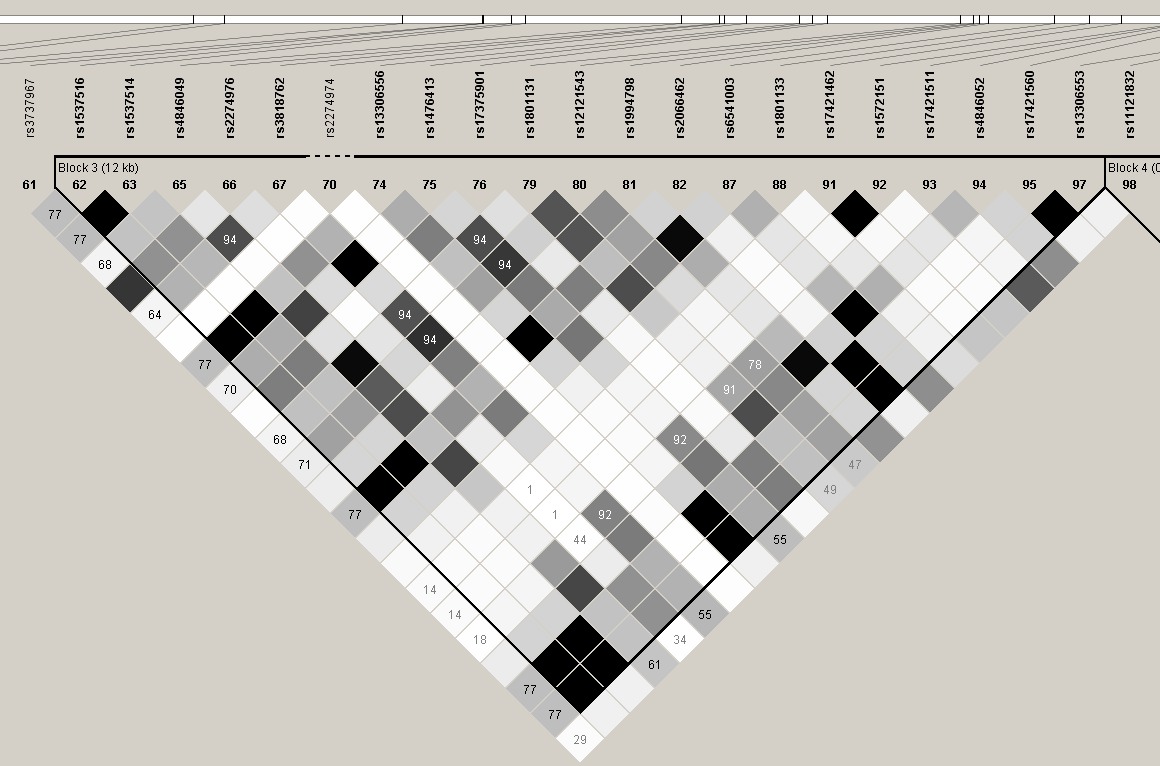


The circled SNP is rs1801131 (*MTHFR* Glu429Ala), which lies in a 12kb LD block. The black squares indicate other highly correlated SNPs (r^2^>0.80).

Figure S2. Linkage disequilibrium block containing the *ERCC5* His46His (rs1047768) polymorphism


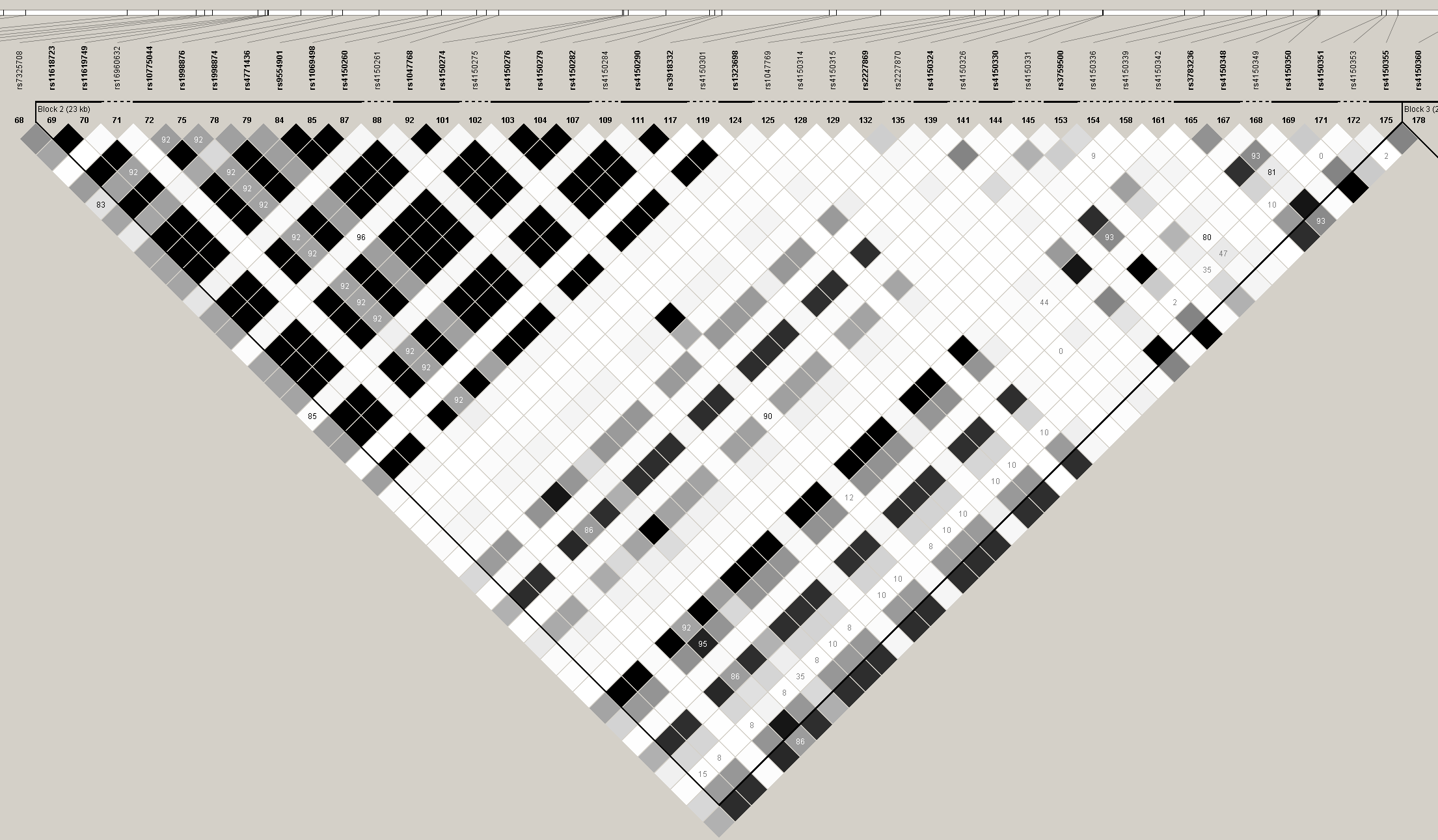


The circled SNP is rs1046678 (*ERCC5* His46His), which lies in a 23kb LD block. The black squares indicate other highly correlated SNPs (r^2^>0.80).

**References for Supplementary Information**
